# Role of Terpenophenolics in Modulating Inflammation and Apoptosis in Cardiovascular Diseases: A Review

**DOI:** 10.3390/ijms24065339

**Published:** 2023-03-10

**Authors:** Muhamad Adib Abdul Ghani, Azizah Ugusman, Jalifah Latip, Satirah Zainalabidin

**Affiliations:** 1 Programme of Biomedical Sciences, Centre of Toxicology and Health Risk Study, Faculty of Health Sciences, Universiti Kebangsaan Malaysia, Kuala Lumpur 50300, Malaysia; 2 Department of Physiology, Faculty of Medicine, Universiti Kebangsaan Malaysia, Kuala Lumpur 56000, Malaysia; 3 Department of Chemical Sciences, Faculty of Science and Technology, Universiti Kebangsaan Malaysia, Bangi 43600, Malaysia

**Keywords:** terpenophenolics, terpenes, phenolics, cardiovascular diseases (CVDs), atherosclerosis, inflammation, apoptosis, oxidative stress

## Abstract

One in every three deaths worldwide is caused by cardiovascular diseases (CVDs), estimating a total of 17.9 million deaths annually. By 2030, it is expected that more than 24 million people will die from CVDs related complications. The most common CVDs are coronary heart disease, myocardial infarction, stroke, and hypertension. A plethora of studies has shown inflammation causing both short-term and long-term damage to the tissues in many organ systems, including the cardiovascular system. In parallel to inflammation processes, it has been discovered that apoptosis, a mode of programmed cell death, may also contribute to CVD development due to the loss of cardiomyocytes. Terpenophenolic compounds are comprised of terpenes and natural phenols as secondary metabolites by plants and are commonly found in the genus *Humulus* and *Cannabis*. A growing body of evidence has shown that terpenophenolic compounds exhibit protective properties against inflammation and apoptosis within the cardiovascular system. This review highlights the current evidence elucidating the molecular actions of terpenophenolic compounds in protecting the cardiovascular system, i.e., bakuchiol, ferruginol, carnosic acid, carnosol, carvacrol, thymol and hinokitiol. The potential of these compounds is discussed as the new nutraceutical drugs that may help to decrease the burden of cardiovascular disorders.

## 1. Introduction

According to the World Health Organization (WHO), cardiovascular diseases (CVDs) are the leading cause of death globally, comprising 17.9 million in 2019 and expected to surge up to 23 million by 2030. CVDs such as heart attack and stroke are responsible for 85% of these deaths. CVDs accounted for 38% of the 17 million premature deaths attributed to noncommunicable diseases (NCDs) in 2019 [1]. As highlighted by the Centers for Disease Control and Prevention (CDC) in 2022, NCDs, mainly CVDs, are a major burden to the US economy, with over $216 billion spent on the healthcare system and $147 billion in losses due to the increasing rate of absenteeism in the workplace and the resulting decreased productivity [2].

CVDs includes many disorders, such as cardiac muscle diseases and vascular dysfunctions that disrupt the blood supply to the heart, brain, and other major organs. The most prevalent causes of CVD mortality and morbidity are ischemic heart disease, stroke, congestive heart failure, and rheumatic heart disease [3]. There are two categories of risk factors contributing to the development of CVDs: modifiable and non-modifiable. Among the modifiable risk factors are smoking, excessive intake of alcohol, lack of physical activity, obesity, and poor diet. Physiological risk factors, such as hypertension, diabetes, and dyslipidemia, also fall within the category of modifiable risk factors [4]. Socioeconomic status (SES) is also one of the modifiable risk factors for CVDs, where more significant socioeconomic disparities and lower levels of income, education, and employment status lead to such issues as less manageable living expenses, higher stress levels, poor dietary habits, and insufficient physical activity [5]. In contrast, non-modifiable risk factors for heart disease include age, gender, ethnic background, and family history.

To date, the use of traditional herbs and plant-derived extracts is emerging as alternative and complementary therapy in preventing and treating CVDs. Among the benefits of using natural compounds as nutraceutical agents are their inexpensive cost and relatively safety [6]. Natural compounds such as terpene exhibited effective pharmacological effects, which could be attributed to their ability to scavenge free radicals, enhance the antioxidant system, and alter redox signaling. Terpene compounds are secondary metabolites primarily found in plants as constituents of essential oils. They are biosynthesized in the mevalonate pathway from C_5_ isoprenoid building blocks that bond sequentially as (C_5_)*_n_* structures. The structure of the isoprenoids is used to classify them into the following groups: hemiterpenes (C_5_), monoterpenes (C_10_), sesquiterpenes (C_15_), diterpenes (C_20_), sesterterpenes (C_25_), triterpenes (C_30_), and tetraterpenes (C_40_) [7]. Other functional groups, such as alcohols, aldehydes, and phenols, may also be used, forming a highly diverse and complex class of secondary metabolites [8]. Terpenophenolic compounds are terpene units consisting of phenol moiety (i.e., aromatic benzene ring with hydroxyl group). Accumulating evidence has shown the role of terpenophenolics in attenuating major risk factors of CVDs, such as high levels of low-density lipoprotein, cholesterol, hyperglycemia, and hypertension [9,10,11]. However, clinical trials with plant extract or plant-derived compounds have centered mostly on cancer and neurological diseases, with limited investigations into their cardioprotective effects. Presently, there are no clinical studies being conducted focusing on the pharmacological effects of terpenophenolic compounds in circumventing CVDs. Hence, this review provides proof of concept for translational medicine of terpenophenolic compounds in preclinical studies.

Considering the significance of plant metabolites as primary compounds and the enormous burden of CVDs, this review aims to present recent findings on the pharmacological activities of terpenophenolic compounds as cardioprotective agents by targeting oxidative stress, inflammation, and apoptosis pathway. The literature search was conducted in the PubMed, Web of Science (WOS) and ScienceDirect databases, which covered the time frame from 2017 to 2022. Search strings used were: terpenophenolic; terpene; terpenoid; heart failure; cardiovascular disease; cardiac dysfunction; atherosclerosis; inflammation; apoptosis; oxidative stress. We summarized the articles and incorporated the literature search according to the search results.

## 2. The Role of Inflammation and Apoptosis in Cardiovascular Diseases

The main culprit of CVDs is the impairment of the vascular system [12]. Vascular dysfunction is characterized by the narrowing of the arterial lumen, activation of platelet, impaired vasomotion and vascular tone, thrombosis, vascular permeability, and vascular fibrosis [13]. The progression of vascular dysfunction is highly associated with hyperglycemia, dyslipidemia, and hypertension. Vascular endothelial cell dysfunction affects not only the heart [14], but also the brain [15] and kidneys [16].

Inflammation has been demonstrated to play a significant role in the onset of vascular dysfunction, especially in the fundamental steps during the pathogenesis of atherosclerosis, coronary vasoconstriction, and myocardial ischemia [17]. Numerous studies have shown the substantial role of inflammation in CVD development, such as atherosclerosis, endothelial dysfunction, myocardial infarction, high blood pressure, and heart failure. In the event of atherosclerosis plaque formation, pro-inflammatory cytokines have been found to modify the low-density lipoprotein (LDL) into more highly oxidized LDL (ox-LDL) molecules and exacerbate the endothelial dysfunction. Other than that, inflammation also triggers the expression of adhesion molecules, such as the intercellular adhesion molecule 1 (ICAM-1), vascular cell adhesion molecule 1 (VCAM-1), and selectins [18]. In the vascular wall, infiltrated monocytes differentiate into macrophages and form foam cells with ox-LDL, then release various pro-inflammatory cytokines such as interleukin (IL)-1, IL-3, IL-8, and IL-18, and tumor necrosis factor alpha (TNF-α) [19,20].

**Figure 1:**
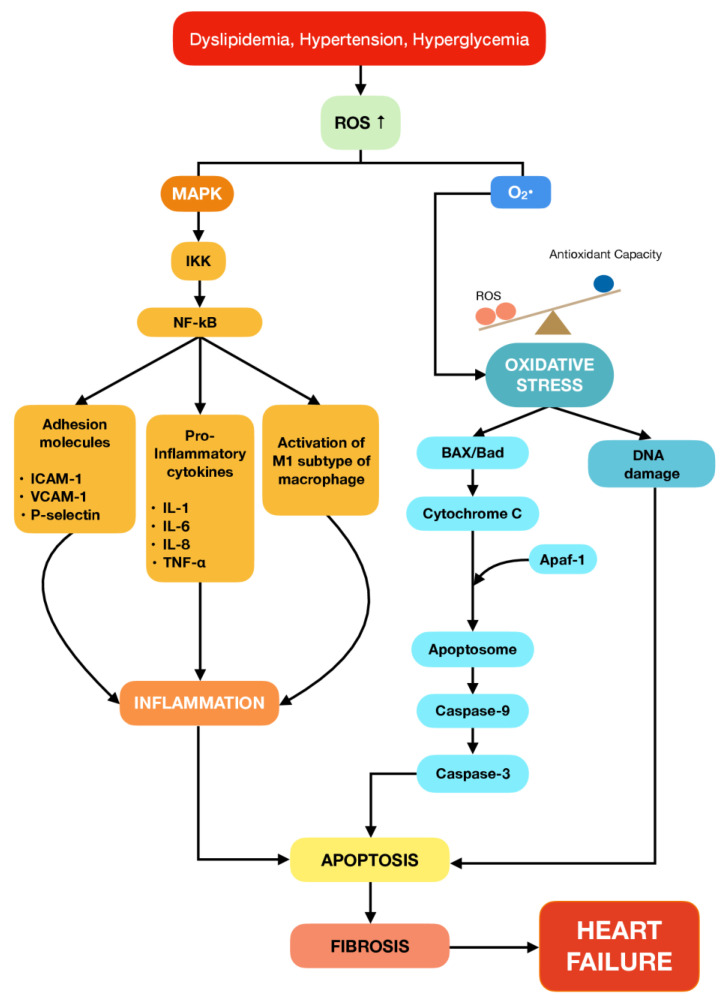
Summary of the signalling pathways involved in the development of heart failure. Hyperlipidemia, hypertension, and hyperglycemia trigger the generation of reactive oxygen species (ROS), which induces oxidative stress in the cardiomyocytes. ROS also induces inflammation responses and cell apoptosis mediated by the mitogen-activated protein kinase (MAPK) pathway. As a compensation mechanism to maintain the structural integrity of the heart, apoptotic cardiomyocytes were degraded and then replaced by myocardial fibrosis. On the contrary, the alteration in the architecture of the heart creates malfunction in both the systolic and diastolic flows, ultimately resulting in heart failure.

The signaling pathway that was found to be responsible for the progression of heart failure mediated by inflammation response and apoptotic factors could be related to the mitogen-activated protein kinase (MAPK) pathway, as summarized in Figure 1 [21,22,23]. Persistent dyslipidemia, hyperglycemia, and hypertension can significantly drive reactive oxygen species (ROS) production and set off inflammatory responses and apoptosis signalling pathways [24,25]. Apoptosis has been widely known to be involved in both acute and chronic loss of cardiomyocytes in ischemia-reperfusion (I/R) injury, myocardial infarction, and progression of heart failure [26,27]. Several studies demonstrated that apoptosis plays a pivotal role in cardiomyocyte loss in human and rodent models with heart failure. In addition, the increase of pro-inflammatory cytokines expression and apoptotic factors have been noted in the cardiotoxicity rat model. Furthermore, previous studies revealed that both extrinsic and intrinsic apoptosis pathways coincide in executing the metabolic changes and morphological structure. The extrinsic pathway involves the activation of death receptors or tumor necrosis factor receptors (TNFR) that are triggered by stimuli such as TNF-α. Meanwhile, the intrinsic pathway involves mitochondria-mediated apoptosis, which is caused by the direct effect of excessive production of ROS in the heart. Nevertheless, both pathways eventually meet up with the same effector caspase, such as caspase-3 [28].

The development of myocardial fibrosis is an adaptive mechanism characterized by a scarring event where apoptotic cells are replaced in the myocardium to preserve the integrity of the heart structure [29]. Dead cardiomyocytes are replaced with fibrotic tissue deposition, including collagen I and collagen III, in the extracellular matrix (ECM) space. Due to the characteristics of stiff collagen tissue, the heart shows an abnormality in its functioning, such as reduced force in contractility as well as myocardial hypertrophy [22]. Uncontrolled myocardial fibrosis leads to the onset of diastolic and systolic dysfunction, which in turn leads to the progression of heart failure.

The loss of cardiomyocytes becomes a determinant of morbidity and mortality following myocardial infarction; hence, minimizing the loss of cardiomyocytes becomes essential in delaying the progression of heart failure. Terpenophenolic compounds have been revealed to alleviate cardiovascular-related complications such as high blood pressure, atherosclerosis, and heart function through modulation of inflammation and apoptosis via the MAPK pathway.

## 3. Terpenes and Their Subclasses

Terpenes, also known as isoprenoids, are secondary metabolites primarily found in plants as constituents of essential oils. Terpenes form a large and structurally diverse family of natural products derived from C5 isoprene units that are joined in a head-to-tail fashion [30]. Terpenes are made up of the isoprene unit (building blocks), and each isoprene unit contains five carbon atoms; one of the carbon atoms is linked by a double bond [31]. Isoprene was identified as a degradation by-product of different natural cyclic hydrocarbons and was proposed as the basic building blocks for these hydrocarbons, also termed “isoprenoids”. Therefore, the classification of terpenes is based on the number of isoprene units (C_5_H_8_)*_n_* present in their molecular structure; i.e., hemiterpenes (C_5_, n = 1), monoterpenes (C_10_, n = 2), sesquiterpenes (C_15_, n = 3), diterpenes (C_20_, n = 4), sesterterpenes (C_25_, n = 5), triterpenes (C_30_, n = 6), and tetraterpenes (C_40_, n = 8) as shown in the Table 1. As these naturally occurring hydrocarbons are progressively isolated and identified, additional subclasses are introduced to the existing classes. The subclasses are outlined according to the number of rings in the structure of terpenes, i.e., acyclic (open structure/no ring), monocyclic (one ring), bicyclic (two rings), tricyclic (three rings), tetracyclic (four rings), as shown in Table 2 [32]. Natural terpenoids follow the simple arrangement of a linear chain of isoprene units from head to tail, such as geraniol (C_10_), farnesol (C_15_) and geranylgeraniol (C_20_). Most of the terpenoids are further transformed by cyclization reactions; however, the arrangement of the isoprene unit from head to tail can be distinguished, such as menthol, bisabolene, and taxadiene. Consequently, the diversity of terpenoid structures has contributed to the re-arrangement of the existing linear arrangement of isoprene units, which is due to the ionization capability of these isoprene units into various types of carbocations. Furthermore, the carbocation re-arrangement is followed by ornamental reactions where other functional groups such as alcohols, aldehydes, ketones, ethers, esters, phenol, and lactones will be added [33], thus “terpenoids” terminology is used instead of “terpenes”, which refers to unsaturated hydrocarbons. Ultimately, terpenoids constitute a group of secondary metabolites that is highly diverse and complicated; in most cases, post-rearrangement reactions give rise to difficulty in isoprene units identification, and there is the possibility that several carbons have been relocated or missing from the isoprene units. In terms of construction, these compounds exhibit high structural diversity related to their wide range of functions and biological activities, making them more attractive as possible drug candidates for preventive and therapeutic purposes [34].

**Figure 2:**
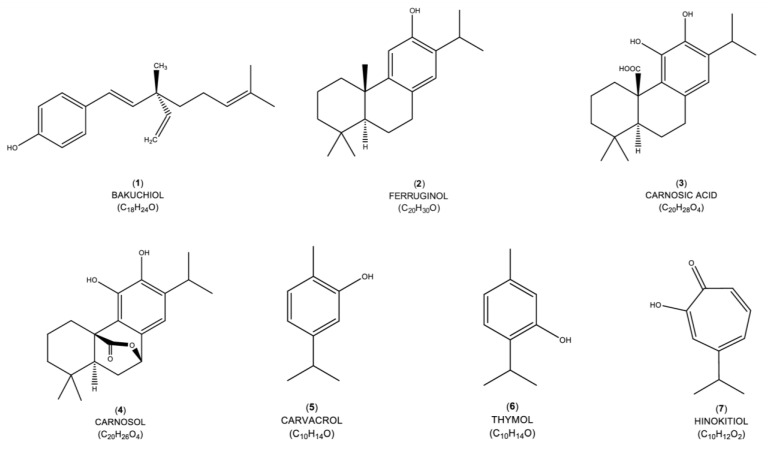
Chemical structures of the terpenophenolic compounds with potential to be used in the development of novel medications to treat cardiovascular diseases.

Terpenes are the most abundant secondary metabolites, with approximately 1000 monoterpenes and 3000 sesquiterpenes identified [35]. However, there are only around 50 phenylpropanes that contain benzene rings. Terpenes are generated via the mevalonic pathway. Meanwhile, phenylpropanes, which have a benzene ring in their molecular structure, are synthesized via the shikimic pathway. The mevalonic pathway depends on mevalonic acid, a chemical intermediate made by the plant. The plant converts mevalonic acid to a five-carbon structure (with the isoprene arrangement), which is the defining feature of all terpenes. Meanwhile, the shikimic pathway uses an enzyme known as phenylalanine ammonia-lyase (PAL) to generate phenolic compounds containing benzene rings [36]. This pathway synthesized terpenophenolic compounds like bakuchiol and ferruginol. Terpenophenolic compound is characterized by a hydroxyl group attached to a benzene ring. Unlike all terpenes whose name ends in “-ene”, this compound name ends in –“ol”. There are only two common terpenophenolic compounds found in essential oils: thymol and carvacrol. Benzene, which is composed of aromatic rings, can be simply derived from aliphatic (non-benzene) rings; on the other hand, the reverse reaction takes place very rarely. Figure 2 represents the chemical structure of terpenophenolic compounds obtained by a search engine that has the potential to function as cardioprotective agents.

Terpenes are widely used in industry as flavour enhancers, scent additives in shampoo and soaps, and products such as disinfectants and detergents [37,38]. Since terpenes are the core constituent that is found in many essential oils, they are also highly valued in aromatherapy. Aromatherapy makes use of terpenes as a constituent due to the distinctive smell they offer. In the world of aromatherapy, terpene is called terpenoids due to the presence of oxygen molecules in their molecular structure [8]. Although terpenoids is not a chemical term, they can be used to differentiate between terpene molecules that consist of oxygen or not. In the pharmaceutical industry, terpenes have shown their potency as new therapeutic agents to treat or prevent cancer, malaria, inflammation, pain, CVDs, and various infectious diseases [39]. In particular, mono- and sesquiterpenes have been used as a complex in delivery systems such as liposomes, nanocapsules and cyclodextrins due to their low solubility and high volatility in the aqueous system [40,41]. Table 3 summarizes the terpenophenolic compounds that exhibit cardioprotective effects.

## 4. Role of Terpenophenolic Compounds on Inflammation and Apoptosis in Cardiovascular Diseases

### 4.1. Bakuchiol

Bakuchiol (**1**) is the main bioactive meroterpene isolated from the seeds and leaves of *Psoralea corylifolia*, a member of the Leguminosae plant family [57]. Indian and Chinese people have traditionally used bakuchiol to treat various diseases [58]. A cell viability assay was conducted on H9c2 cells exposed to bakuchiol at a dose ranging from 2–10 μM and the results showed no toxicity effects [42]. In 2020, a study revealed the potential of bakuchiol as a cardioprotective agent for improving heart function in hyperglycemia-induced diabetic cardiomyopathy rats. Bakuchiol alleviated the decrease of ejection fraction (EF) and fractional shortening (FS) of the left ventricle and increased end-systolic and end-diastolic volume. Bakuchiol regulated the redox status by facilitating the suppression of silent information regulator 1 (SIRT1) and nuclear factor erythroid 2-related factor 2 (Nrf2) gene expression, which are the primary factors in producing antioxidant enzymes. Bakuchiol remarkably aggravated the superoxide dismutase (SOD) and glutathione peroxidase (GSH-Px) production and reduced the production of reactive oxygen species (ROS) in diabetic myocardium tissue [42]. Moreover, bakuchiol protected the heart from hypertrophy by modulating the hypertrophy markers, i.e., atrial natriuretic peptide (ANP), alpha-myosin heavy chain (α-MHC), brain natriuretic peptide (BNP), and beta-myosin heavy chain (β-MHC). In addition, bakuchiol also mitigated the increase of ANP, BNP and β-MHC gene and protein expression, and significantly increased the α-MHC protein level. Histological findings of cross-sectional left ventricle myocytes have shown that bakuchiol attenuated the increase of cardiomyocyte size. Another study by Wang et al. [43] corroborated the latest findings that bakuchiol possessed an anti-hypertrophic effect in neonatal rat cardiomyocytes (NRCM) induced by angiotensin II (Ang II). Other than that, the authors also revealed that bakuchiol improved cardiac dysfunction and reduced the enlargement of cardiomyocytes induced by pressure-overload.

Furthermore, bakuchiol alleviated myocardial fibrosis by limiting the collagen 1 & 3 depositions in the diabetic myocardium cells. In addition, bakuchiol inhibited the gene expression level of α-SMA and suppressed mothers against decapentaplegic 3 (Smad3), which played a role in the development of fibrosis. In a study where H9c2 cells were induced by a high glucose concentration with the presence of SIRT1 inhibitor, it was revealed that bakuchiol reversed its pharmacological effects. Hence, the author postulated that bakuchiol exerts its cardioprotective effect via activation of the SIRT1 pathway [42].

In overload pressure-induced hypertrophy in rats, bakuchiol modulates the inflammatory response by suppressing the release of pro-inflammatory cytokines: tumor necrosis factor alpha (TNF-α), interleukin-6 (IL-6), and monocyte chemoattractant protein-1 (MCP-1) [43]. Protein expression of phosphorylated IkappaB kinase (IKKβ), IKBα, and p65 levels increased in the hypertrophied rat induced by overload pressure; however, bakuchiol attenuated these changes. The author postulated that the nuclear factor kappa B (NF-κB) pathway is possibly the main culprit in mediating cardiac hypertrophy. An in vitro study using NRCM in the presence of Ang II corroborated the finding in an in vivo study that bakuchiol suppressed the activation of the NF-κB pathway. PDTC, a selective NF-κB inhibitor, was used in NRCMs with Ang II and has shown that enlargement of cardiomyocytes was blocked. Wang et al. [43] concluded that bakuchiol’s anti-hypertrophy effect largely depends on inhibiting the NF-κB pathway.

### 4.2. Ferruginol

Ferruginol (**2**) is a naturally occurring diterpenoid that is mostly found in the *Salvia* plant species [59]. A toxicity study found that exposure to ferruginol at a higher concentration of 5 μM had a deleterious effect on the H9c2 cells. Despite that, ferruginol at a dose varying from 0.1–0.5 μM gives optimum therapeutic effect on H9c2 cells. Ferruginol effectively attenuated myocardial injury caused by overdosed isoprenaline hydrochloride by reducing cardiac injury markers: cardiac troponin-T (cTnT), cardiac troponin-I (cTnI), creatine kinase (CK), and creatine kinase-MB (CKMB) in myocardial infarction model [44]. This finding supports the latest study by Li et al. [45] that demonstrated that ferruginol reduced cardiac injury markers in the doxorubicin (DOX)-induced cardiotoxicity model. Furthermore, ferruginol exerted its antioxidant properties in the reduction of lipid peroxidation markers and malondialdehyde (MDA) and upregulated the antioxidant enzymes SOD, GPx, catalase (CAT), and glutathione (GSH). Induction of overdosed isoprenaline caused alteration in sodium-potassium pump (ATPases) activity, which is associated with contraction and relaxation of cardiac muscle. However, ferruginol was capable of restoring ATPases activity and revive mitochondrial function as it may protect against oxidative stress by inhibiting membrane lipid peroxidation [44].

Moreover, ferruginol attenuated inflammation by reducing the release of inflammatory cytokines, namely TNF-α, IL-6, and NF-κB. This result is supported by the histoarchitecture of cardiomyocytes, where ferruginol significantly prevented intense cardiac fiber with inflammatory cells [44]. In addition, observation under a transmission electron microscope demonstrated that attenuated the loose arrangement of the mitochondrial and a significant reduction in the number of mitochondria caused by DOX treatment [45]. To support these findings, an in vitro study has shown that the decreased adenosine triphosphate (ATP) content in DOX treatment was mitigated by ferruginol. A study by Ulubelen [60] discovered that ferruginol exhibits the same antihypertensive effect as propranolol (beta-blocker) by decreasing the systolic blood pressure, mean arterial pressure (MAP) and heart rate. Besides, ferruginol improved ventricular function by alleviating the decrease in ejection fraction (EF) and fractional shortening (FS) in DOX-induced cardiotoxicity [45].

In hypoxia/reoxygenation (H/R)-subjected H9c2 cardiomyoblasts, pretreatment of ferruginol was able to preserve cell viability and reduce the percentage of apoptotic cells. It also reduced cardiac injury and the production of ROS. Li et al. [45] suggested that ferruginol exerts cardioprotective effects against DOX-induced cardiotoxicity by preserving the mitochondrial from the production of ROS, limiting damage to heart function and attenuating the apoptotic process by upregulating mRNA level of SIRT1 and peroxisome proliferator-activated receptor-gamma coactivator-1alpha (PGC-1α).

### 4.3. Carnosic Acid

Carnosic acid (**3**) is a naturally occurring diterpenoid compound mainly found in rosemary (*Rosmarinus officinalis*), Greek sage (*Salvia fruticosa*), and holy basil (*Ocimum sanctum*). All these plants are members of the Lamiaceae family and are native to the Mediterranean region [61]. A cell viability assay has been conducted on the H9c2 cells exposed to the carnosic acid ranging from 0.1 μM to 10 μM, and it has been shown to exert a protective effect in a dose-dependent manner [62]. In 2018, Lee et al. [46] revealed that carnosic acid exhibits an anti-obesity effect by lowering body weight and the percentage of fat tissues in ovariectomized rats with a high-fat diet. In addition, carnosic acid reduced the elevated levels of the hormone leptin and insulin, as well as total glycerides and free fatty acids. Moreover, carnosic acid attenuated lipogenic genes, sterol regulatory element-binding protein-1c (SREBP1c), and fatty acid synthase (FAS), which were elevated in ovariectomized with high-fat diet rats. Following that, carnosic acid also decreased the fatty acid synthesis and stimulated β-oxidation genes by increasing the messenger RNA (mRNA) level of peroxisome proliferator-activated receptor gamma (PPAR-γ) and carnitine palmitoyltransferase 1 (CPT-1).

Furthermore, in ovariectomized and high-fat diet rats, there is an increasing trend in the gene expression level of adipocyte differentiation and fat accumulation associated genes in white adipose tissue (WAT), PPAR-γ, adipocyte protein 2 (aP2) and lipoprotein lipase (LPL); interestingly, carnosic acid was able to restore all these changes. Histological observation of WAT supported the biochemical findings whereby carnosic acid markedly ameliorated the increase of adipocyte sizes. Carnosic acid also reduced the inflammatory mediators TNF-α and IL-6 that were raised in white adipose tissue in the ovariectomized with high-fat diet rats [46].

A study by Wei et al. [47] demonstrated that carnosic acid exerted its cardioprotective effect by alleviating the progression of cardiac hypertrophy in overload pressure-induced myocardial hypertrophy in rats. Carnosic acid reduced the rise of protein and gene expression of hypertrophic markers, i.e., ANP, BNP and β-MHC. In addition, microscopic observation found that carnosic acid mitigated the enlarged cardiomyocyte area induced by pressure overload. Besides that, carnosic acid also exhibited antioxidant properties by regulating the redox status, increasing the SOD levels, and suppressing the MDA and nicotinamide adenine dinucleotide phosphate (NADPH) activity. Carnosic acid also reduced the protein and gene expression of NAPDH oxidase 2 (NOX 2) and NOX 4, which played a role in the generation of free radicals. Previous evidence by Zhang et al. [48] showed consistency in the role of carnosic acid in alleviating oxidative stress in DOX-induced cardiotoxicity in rats. The author postulated that carnosic acid possessed antioxidant properties by activating the Nrf2 pathway. Furthermore, both authors demonstrated that carnosic acid improved cardiac function by elevating the EF of the left ventricle.

Carnosic acid plays a role in the modulation of the inflammation response in DOX-induced cardiotoxicity rats by reducing the release of pro-inflammatory cytokines: nitric oxide, TNF-α, IL-6, and COX-2 [48]. H9c2 cells treated with DOX revealed that carnosic acid suppressed the increased protein expression of p-NF-κB, IL-1β and IL-18. Moreover, carnosic acid was able to regulate the apoptosis and autophagy signalling pathways by upregulation of B-cell lymphoma 2 (Bcl-2) protein, downregulation of cleaved caspase-3 protein, and suppressing the autophagy associated-molecules: light chain 3B II (LC3BII), autophagy-related genes-5 (ATG-5), and ATG-7. In addition, carnosic acid also regulates the apoptotic protein in DOX-treated H9c2 cells.

### 4.4. Carnosol

Carnosol (**4**) is a naturally occurring diterpenoid compound and one of the main components of the extract of *Rosmarinus officinalis* L., a woody perennial herb, that belongs to the Lamiaceae family. This plant has been used as a traditional remedy for alleviating headaches, stomach aches, and rheumatic pain [63]. In the laboratory setting, carnosol has been proven to exert a cardioprotective effect by increasing the cell viability of H9c2 cardiomyoblast cells in the presence of lipopolysaccharides (LPS) [49]. This result is consistent with a previous study by Ou et al. [50], which revealed that carnosol improved the viability of rats’ multipotent progenitor cells (MAPCs) induced by hydrogen peroxide (H_2_O_2_).

Carnosol ameliorated the decrease of protein expression of Nrf-2 induced by H_2_O_2_ and regulated redox status by upregulating the GSH, glutathione S-transferases (GST) and catalase (CAT) levels and suppressing the excessive production of ROS. A study by Li et al. [64] also indicated that carnosol increased the gene expression of Nrf-2 and cytoprotective proteins such as heme oxygenase-1 (HO-1) and endothelial nitric oxide synthase (eNOS) in human microvascular endothelial cells (HMVECs). Carnosol significantly alleviated the decrease of the endothelial differentiation markers: octamer-binding transcription factor-4 (OCT-4), fetal liver kinase-1 (Flk-1), and cluster of differentiation 31 (CD-31) caused by H_2_O_2_. Lack of these markers leads to failure of migration and proliferation of endothelial progenitor cells to the injury site for endothelial repair, which results in cardiac dysfunction.

Furthermore, carnosol exhibits anti-inflammatory properties by downregulating the pro-inflammatory cytokines, TNF-α, IL-1β, IL-6 and COX-2 in H9c2 cells induced by LPS. The author postulated that carnosol exerted its anti-inflammatory effect in LPS-induced H9c2 by inhibiting the NF-κB pathway [49]. Moreover, carnosol displayed an anti-apoptosis effect by downregulating the proapoptosis protein and caspase-3 activity and reducing the apoptotic percentage in MAPCs induced by H_2_O_2_.

### 4.5. Carvacrol

Carvacrol (**5**) is a monoterpenoid compound which is abundant in the essential oils of aromatic plant species, including *Origanum*, *Thymus*, and pepperwort [65]. It is most prevalent in *Nigella sativa* L., an annual flowering plant with green-to-blue flowers and black seeds that is a member of the Ranunculaceae family [66]. A toxicology study using the caco-2 cell line derived from human colorectal carcinoma revealed a toxic effect of carvacrol dependent on time and concentration. Exposure to carvacrol with a dose of 115 μM for 24 h on the cell line did not give any cytotoxic effect. However, as the dose increases, a shorter time is taken to alter the morphology of the cells [67]. Carvacrol at a lower concentration exhibited a protective effect on the cell against H_2_O_2_ damage. In addition, an in vivo study revealed that a combination of carvacrol and thymol at (10–20 mg/kg/body weight) or higher concentration did not adversely affect Wistar-Albino testes and kidneys [68]. In 2017, Chen et al. [51] revealed that carvacrol exhibits a cardioprotective effect against myocardial I/R injury by suppressing electrocardiogram ST segment elevation and reducing infarcted volume in a dose-dependent manner. In another study, carvacrol was found to restore blood pressure to the normal level [52]. In response to myocardial I/R stress, carvacrol attenuated lipid peroxidation by reducing the MDA levels [51]. In DOX-induced cardiotoxicity model, carvacrol alleviated the release of cardiac injury markers: lactate dehydrogenase (LDH), aspartate aminotransferase (AST), CK-MB, and troponin-I and significantly increased the antioxidant enzymes: GSH, SOD and CAT [69].

Moreover, carvacrol demonstrated anti-apoptosis activity by alleviating the percentage of apoptotic cells in myocardial I/R injury. These findings were corroborated by an in vitro study that showed that carvacrol dose-dependently increased the cell viability and attenuated apoptosis in H9c2 cardiomyoblast induced by H/R injury [51]. Sadeghzadeh et al. [52] proved that carvacrol modulated apoptosis signalling pathways by upregulating the gene expression of Bcl-2 and Bcl-xL, and downregulating gene expression of Bax and Bad in hypertrophied rat hearts. In H/R-induced H9c2 cells, carvacrol increased the protein expression of Bcl-2, and suppressed the protein expression of Bax and cleaved caspase-3. Histoarchitecture preservation of cardiomyoblast strengthened the evidence that carvacrol could protect the heart by preventing the development of collagen deposition in hypertrophied hearts [52]. Chen et al. [51] postulated that carvacrol mediated its cardioprotection by modulation of apoptosis signalling via the MAPK/ERK pathway, where carvacrol downregulated protein expression of phosphorylated-p38 (p-p38) and phosphorylated c-Jun N-terminal kinase (p-JNK) and upregulated phosphorylated extracellular signal-regulated kinase (p-ERK).

### 4.6. Thymol

Thymol (**6**) is a natural monoterpenoid compound and one of the primary active constituents in the essential oil of *Thymus vulgaris*, a flowering plant belonging to the Lamiaceae family [70]. In the pharmaceutical industry, thymol has been used as an active ingredient in mouthwash to kill bacteria. According to the Environmental Protection Agency, thymol has no known toxic effects when used in animals and humans. The United States Food and Drug Administration has classified thymol as Generally Recognized As Safe (GRAS) for use as a food additive; thus, it is deemed safe with little toxicity [71]. In a preclinical setting, thymol has shown the potential to be a cardioprotective agent by exerting its biological activities in the modulation of lipid profiles of hypercholesterolemic rats [53]. Thymol suppresses the increment of total cholesterol and oxidized LDL, which is attributed to the progression of atherosclerosis. Moreover, thymol has been revealed to prevent the development of atherosclerosis by suppressing inflammatory mediators: IL-1β, IL-6, TNF-α, and TNF-β and adhesion molecules: vascular cell adhesion molecule-1 (VCAM-1), MCP-1, and matrix metalloproteinase 9 (MMP-9) in hyperlipidemic rabbits. Histological observation was in parallel with the biochemical data, whereby thymol was shown to reduce the lipid lesion in the intimal surface of the thoracic aorta [72]. In hypercholesterolemia-induced oxidative stress, thymol attenuated the lipid peroxidation marker, MDA and significantly increased GPx. Another study by El-Marasy et al. [54] supported these findings; thymol was able to improve antioxidant status in an adrenaline-induced myocardial injury model. Following that, thymol decreased the release of tissue injury markers: LDH, AST and CK. Histoarchitecture analysis revealed that thymol prevented the alteration of cardiomyocyte structure in a dose-dependent manner.

El-Marasy et al. [54] has proven that thymol can improve heart function by increasing the heart rate to the normal level, ameliorating the ST segment elevation and RR intervals in adrenaline-induced myocardial injury. The author proposed that thymol protects the heart by acting as an anti-inflammatory, anti-apoptosis, and antioxidant agent. Thymol suppressed the release of pro-inflammatory cytokines, NF-κB, and IL-1β and pro-apoptotic protein, activated caspase-3 and increased the protein expression of Bcl-2. The outcome is consistent with previous studies by Bayatmakoo et al. [53], whereby thymol decreased apoptosis on rat carotid tissue induced by hypercholesterolemia via upregulation of protein expression of Bcl-2 and downregulation of caspase-3 and phosphorylated-p38.

### 4.7. Hinokitiol

Hinokitiol (**7**) or also known as β-thujaplicin is a monoterpenoids compound that constitutes a volatile oil that is abundantly found in *Thujopsis dolabrata* belonging to the cypress family. This plant, also named *Hiba arborvitae*, is a dense, slow-growing, pyramidal, evergreen conifer native to the humid forested regions of central Japan [73]. In industries, hinokitiol has been widely used as an active ingredient in hair tonics, toothpaste, cosmetics, and food as an antimicrobial agent [74]. Cell viability assay has been tested on hinokitiol concentrations varying from 2 to 12 μM, and the result showed no cytotoxicity on SEVC 4–10 endothelial cells and A7r5 VSMC. Nonetheless, when the concentration was greater than 24 μM, toxicity was detected in both cell lines, and 80% of the cells reported were alive [55]. Hinokitiol dose-dependently exhibits its anti-atherogenic effect by ameliorating the release of adhesion molecules: sICAM-1, sVCAM-1, and E-selectin in LPS-induced inflamed endothelial cells [55,56]. Increased circulating adhesion molecules will contribute to the progression of atherosclerosis, which manifests as CVDs such as coronary heart disease. Besides, hinokitiol played a role in the modulation of gene related to the progression of atherosclerosis; MMP-2 and MMP-9. In DHEP-exposed vascular smooth muscle cells (VSMC), the gene and protein expression of MMP-2 and MMP-9 were increased, and hinokitiol successfully inhibited those markers.

In H_2_O_2_-induced oxidative damage in the human cardiomyocyte cell line (AC16), hinokitiol markedly limited the inhibition of cell proliferation in a dose-dependent manner. Histological examination revealed that the AC16 cells became round and shrunk after exposure to H_2_O_2_, and hinokitiol prevented those morphological changes [56]. Other than that, it attenuated apoptosis in H_2_O_2_-exposed AC16 cells by 13.12% reduction. Hinokitiol also prevented the AC16 cells from a decrease in the number of cells with condensed chromatin and the formation of apoptotic bodies.

Besides that, hinokitiol is responsible for attenuating the autophagy flux by remarkably decreasing the LC3B-II/I ration and Beclin-1 protein, while increasing the level of p62 [65]. In silico study further corroborated the evidence in the preclinical setting. The molecular docking analysis of hinokitiol revealed that it bonded to the molecular GSK-3β pathway through its phosphorylated-Ser9 site to mediate the autophagy flux. In addition, hinokitiol exerted an anti-apoptosis effect by significantly increasing p21 protein in H_2_O_2_-induced AC16 cells.

## 5. Prospective of Terpenophenolics Compound Playing a Critical Role in Cardiovascular Diseases

Excessive reactive oxygen species and oxidative stress is the main culprit contributing to heart failure development. Nowadays, combating oxidative stress has been popular in the research field, and many researchers are interested in exploring nutraceutical agents as a potential intervention in the oxidative stress mechanism. Terpenophenolic compounds are the potential candidates due to their characteristics as potent antioxidants. Recent preclinical studies have collectively demonstrated the optimistic direction for future clinical studies and revealed that terpenophenolic compounds might have promising potential for the treatment of cardiovascular diseases.

Administrating terpenophenolic compounds as a treatment for cardiovascular research is still new. To date, there is still no current clinical trial using terpenophenolic compounds to explore their potentials effects in circumventing cardiovascular diseases. However, some of the terpenophenolic compounds such as bakuchiol, carvacrol, and thymol are undergoing clinical trial for treating wrinkles and photoaging [75], COVID-19 acute respiratory distress syndrome [76], and obesity [77]. This clearly indicate their high potency as antioxidants and anti-inflammatory agents.

## 6. Conclusions

**Figure 3:**
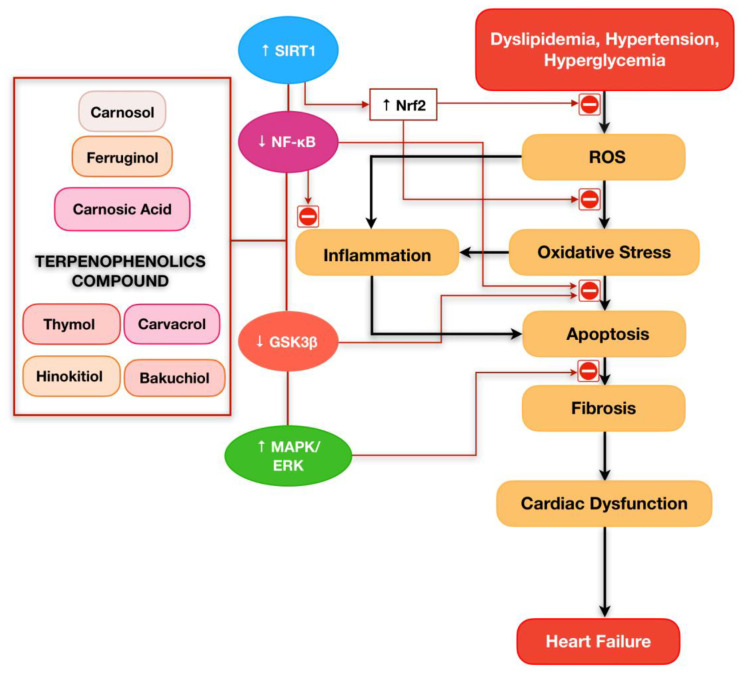
Terpenophenolic compounds exert their potential in attenuating the development of heart failure owing to their ability to counteract reactive oxygen species, oxidative stress, inflammation, apoptosis, and fibrosis via several molecular pathways.

In this review, we have summarised the evidence on the potential pharmacological activities of terpenophenolic compounds in regulating inflammation and apoptosis associated with CVDs. Treatment of various classes of terpenophenolic compounds has been shown effective in preventing and limiting the progression of heart failure (Figure 3). In addition, all terpenophenolics seem to be potent antioxidants, which are proven to upregulate the Nrf2 pathway and increase the endogenous antioxidant level. These may be due to their chemical structures, which consist of a hydroxyl group attached to the aromatic benzene rings. Although numerous preclinical studies have revealed the cardioprotective potential of terpenophenolic compounds, there is no clinical evidence of terpenophenolic compounds documenting their efficacy in the treatment of CVDs. Moreover, the underlying mechanisms driving the pharmacological effects of terpenophenolic compounds on inflammation and apoptosis in the treatment of cardiovascular complications are still uncertain. Therefore, additional research and clinical trials are required to corroborate the efficacy of terpenophenolic compounds as a potential treatment of CVDs.

## Figures and Tables

**Table 1:** Classification of Terpenes.

Terpene	Number of Isoprene Units	Number of Carbon Atoms
Hemiterpenes, C_5_H_8_	1	5
Monoterpenes, C_10_H_16_	2	10
Sesquiterpenes, C_15_H_24_	3	15
Diterpenes, C_20_H_32_	4	20
Sesterterpenes, C_25_H_40_	5	25
Triterpenes, C_30_H_48_	6	30
Tetraterpenes, C_40_H_64_	8	40
Polyterpenes	Many	<40

**Table 2:** Sub-classification of Terpenes.

Molecular Structure	Name
Chain, no ring	acyclic
One ring	cyclic
Two rings	bicyclic
Three rings	tricyclic
Four rings	tetracyclic

**Table 3:** Summary of cardioprotective effects exerted by terpenophenolic compounds published between 2017 and 2022.

Terpenophenolics	Terpenophenolics Subclasses	Study Design	Dose	Findings	Conclusion	References
Bakuchiol	Meroterpenoids	In vivo & in vitro; C57BL6 male mice (20–25 g) & H9c2 cells	60 mg/kg/day; 2, 5, 10 μM	- Bakuchiol inhibited the synthesis of fibrosis-associated protein in diabetic myocardium by reducing gene expression of α-SMA and Smad3. - Bakuchiol ameliorated oxidative stress in diabetic myocardium by increasing SOD, and GSH-Px. - Bakuchiol attenuated cardiomyocyte apoptosis in high glucose-treated H9c2 cells by decreasing TUNEL-positive nuclei and apoptotic ratio.	Protective effect of bakuchiol in limiting the synthesis fibrosis, preventing oxidative damage and cell death in diabetic myocardium may be via the SIRT1 and Nrf2 signalling pathway.	[42]
		In vivo & in vitro; C57BL/6J mice & NRCM cells	10 mg/kg/day; 1, 5, 10 μM	- Bakuchiol ameliorated the cardiomyocyte hypertrophy by reducing gene expression of ANP, BNP and β-MHC and inhibited the enlargement of cardiomyocytes in NRCM. - Bakuchiol ameliorated the cardiomyocyte fibrosis and inflammatory responses by reducing gene expression of collagen 1 & 3 and CGTF, protein and gene expression of TNF-α, IL-6 and MCP-1. - Bakuchiol treatment showed no significant difference in the fraction of apoptotic cells compared to the control group.	Bakuchiol exerts antihypertrophy effects by modulating synthesis of fibrosis and inflammatory responses may be via the NF-κB pathway.	[43]
Ferruginol	Diterpenoids	In vivo; Wistar rats	50 mg/kg/day	- Ferruginol attenuated cardiac injury in myocardial infarction by reducing cTnT, cTnI, CK, CK-MB. - Ferruginol ameliorated oxidative damage in myocardial infarction by reducing malondialdehyde and improving SOD, GPx, CAT and GSH. - Ferruginol attenuated inflammation in myocardial infarction by limiting the secretion of TNF-α, IL-6, and NF-κB.	Cardioprotective effect of ferruginol against myocardial infarction via modulation of inflammatory response and upregulation of antioxidant enzymes.	[44]
		In vivo & in vitro; C57BL/6 mice (20 ± 2) & H9c2 cells	20 mg/kg/day; 0.1 μM	- Ferruginol attenuated the reduced ejection fraction and fractional shortening induced by DOX treatment. - Ferruginol ameliorated the tissue injury marker by reducing the level of LDH and CK-MB caused by DOX treatment. - DOX caused the increase of ROS production, while ferruginol mitigated it. - TUNEL assay demonstrated that DOX caused increasing in apoptotic cells meanwhile ferruginol alleviated the progression of apoptosis.	The cardioprotective action of ferruginol is proven by preserving the mitochondrial from the production of ROS, limiting damage to heart function and attenuating the apoptotic process, possibly via the SIRT1 pathway that mediates mitochondrial biogenesis and fatty acid oxidation.	[45]
Carnosic Acid	Diterpenoids	In vivo; C57BL/6 mice	0.02%	- Carnosic acid diminished the gene expression of adipocyte differentiation and fat accumulation in white adipose tissue by reducing aP2, PPAR-γ and LPL. - Carnosic acid ameliorated inflammation in white adipose tissue by reducing TNF-α, and IL-6. - Carnosic acid reduced the size of abdominal fat adipocytes.	Carnosic acid exhibit antiobesity effect by improving hormone homeostasis and reduced genes expression of liver lipogenesis possibly via the PPAR-γ pathway.	[46]
		In vivo; C57BL/6 mice	50 mg/kg	- Carnosic acid alleviates cardiac hypertrophy by reducing gene expression ANP, BNP, and β-MHC and limiting the enlargement of the cardiomyocyte area. - Carnosic acid attenuated the development of cardiac fibrosis by reducing gene expression of Col 1 & 3, CTGF and α-SMA. - Carnosic acid suppressed the oxidative damage by increasing the SOD level and decreasing the MDA level and NAPDH activity. Protein and gene expression of NOX 2 & 4 are also reduced by carnosic acid. - Carnosic acid inhibited myocardial apoptosis by upregulating Bcl-2 and downregulating Bax and caspase-3.	Cardioprotective of carnosic acid against myocardial remodelling by modulation oxidative stress and apoptosis via the AKT/GSK3β/NOX 4 signalling pathway.	[47]
		In vivo & in vitro; C57BL/6 mice & H9c2 cells	5 mg/kg; 10 μM	- Carnosic acid ameliorated cardiac injury in cardiotoxicity rats by reducing CK-MB and remarkably preventing the enlargement of cardiomyocytes. - Carnosic acid attenuated the myocardial hypertrophy in cardiotoxicity rats by reducing the gene expression of ANP, BNP and β-MHC. - Carnosic acid mitigated the oxidative damage in cardiotoxicity rats by upregulating the antioxidant enzymes; SOD, CAT and GSH and downregulating the lipid peroxidation (MDA). - Carnosic acid suppressed inflammation in cardiotoxicity rats by reducing protein expression of p-NF-κB, IL-1β and IL-18. - Carnosic acid inhibited myocardial apoptosis and autophagy in cardiotoxicity rats by upregulating Bcl-2 and downregulating caspase-3. Autophagy associated-molecules; LC3BII, ATG-5, and ATG-7 suppressed by carnosic acid.	Carnosic acid protects the heart against toxicity by suppression of oxidative damage, inflammation, apoptosis, and autophagy.	[48]
Carnosol	Diterpenoids	In vitro; H9c2 cells	5, 10, 20 μM	- Carnosol ameliorated inflammation in H9c2 cells by reducing protein and gene expression of TNF-α, IL-1β, IL-6 and COX-2.	The protective effect of carnosol against inflammation in the cardiomyoblasts may be via the NF-κB signalling pathway.	[49]
		In vitro; MAPC cells	0.2 μM	- Carnosol inhibited the production of ROS and apoptosis by downregulating caspase-3 activity. - Carnosol limit oxidative damage by upregulating the antioxidant enzyme, GSH, GST, and CAT and protein expression of Nrf-2.	Carnosol promotes vascular health by regulating redox status and downregulating inflammation and apoptosis.	[50]
Carvacrol	Monoterpenoids	In vivo & in vitro; Wistar rats & NRCM cells	25, 50, 100 mg/kg; 0.6 mM	- Carvacrol significantly reduced the infarct volume in myocardial I/R injury rats and suggested that it was dose-dependent. - Carvacrol alleviated the lipid peroxidation marker, MDA in myocardial I/R injury rats. - Carvacrol attenuated the apoptosis protein in mitochondrial injury induced by I/R by upregulating Bcl-2 protein levels and downregulating Bax and cleaved-caspase protein levels.	Carvacrol was found to possess cardioprotective properties, which may be related to its antioxidant and antiapoptotic properties in myocardial I/R injury through activation of MAPK/ERK and Akt-eNOS signalling pathways.	[51]
		In vivo; Wistar rats	5, 10, 25, 50 mg/kg	- Carvacrol improved the MAP in the myocardial hypertrophy group in a dose-dependent manner. - Carvacrol ameliorates apoptosis in myocardial hypertrophy by upregulating gene expression of Bcl-xL and Bcl-2 and downregulating Bad.	Protective effect of carvacrol against myocardial hypertrophy by improving blood pressure and inhibiting apoptosis via regulation of the Bcl-2 family protein.	[52]
Thymol	Monoterpenoids	In vivo; Wistar rats	24 mg/kg	- Thymol attenuated the lipid profile in hypercholesterolemic rats by improving total cholesterol and oxidized LDL. - Thymol mitigated oxidative damage by upregulating the antioxidant enzymes; GSH-Px and TAC and limiting the lipid peroxidation. - Thymol ameliorated apoptosis in hypercholesterolemic rats by reducing protein expression of caspase-3 and phospho-p38 and upregulating Bcl-2 protein.	Thymol preserves carotid tissue by reducing apoptosis and inflammation, which may be a result of its direct antioxidant properties.	[53]
		In vivo; Albino Wistar rats	15, 30, 60 mg/kg	- Thymol ameliorated cardiac injury in myocardial infarction rats by reducing CK levels and improving cardiomyocyte structure in a dose-dependent manner. - Thymol attenuated oxidative damage in myocardial infarction rats by upregulating antioxidant enzyme GSH and limiting the lipid peroxidation. - Thymol mitigated inflammation in myocardial infarction rats by reducing protein expression of NF-κB and IL-1β in a dose-dependent manner. - Thymol attenuated apoptosis in myocardial infarction rats by reducing protein expression of caspase-3 and enhancing Bcl-2 protein.	Thymol has been revealed to have a cardioprotective effect against myocardial infarction via modulating oxidative stress, inflammation, and apoptosis.	[54]
Hinokitiol	Monoterpenoids	In vitro; SEVC4-10 cells and A7r5 cells	4, 12 μM	- Hinokitiol mitigated the inflammation-induced cell adhesion molecules in SEVC4-10 cells by reducing protein expression of E-selectin, sICAM-1, and sVCAM-1. - Hinokitiol inhibited the promotion of atherosclerosis by reducing protein expression of MMP-2 and MMP-9.	Hinokitiol exerts a protective effect against atherosclerosis through modulating cell adhesion molecules and members of the matrix metalloproteinase (MMP) family.	[55]
		In vitro; AC16 cells	20 μM	- Hinokitiol mitigated autophagy flux in cardiomyocytes from oxidative damage by decreasing LC3B-II/I ration, enhancing p62 protein and reducing Beclin-1 protein. - Hinokitiol attenuated apoptosis in cardiomyocytes from oxidative damage by upregulating protein expression of p21. - Molecular docking of hinokitiol found that it inhibited the GSK3β signalling pathway through its phosphorylated at the Ser9 site.	Hinokitiol protects cardiomyocytes from oxidative damage by regulating apoptosis and autophagy, probably through the GSK3β signalling pathway.	[56]

## Data Availability

No new data were created or analyzed in this study. Data sharing is not applicable to this article.

## Abbreviations

AKT	Serine/threonine kinase
ANP	Atrial natriuretic peptide
aP2	Adipocyte protein 2
ATG-5	Autophagy-related genes-5
Bad	Bcl-2 associated death
Bax	Bcl2-associated X protein
Bcl-2	B-cell leukemia/lymphoma 2
Bcl-xL	B-cell lymphoma-extra large
BNP	Brain natriuretic peptide
CAT	Catalase
CGTF	Connective tissue growth factor
CK	Creatine kinase
CK-MB	Creatine kinase-MB
Col 1	Collagen 1
COX-2	Cyclooxygenase-2
cTnI	Cardiac troponin I
cTnT	Cardiac troponin T
DOX	Doxorubicin
E-selectin	Endothelial-selectin
eNOS	Endothelial nitric oxide synthase
ERK	Extracellular signal-regulated kinase
GSH	Glutathione
GSH-Px	Glutathione peroxidase
GSK3β	Glycogen synthase kinase 3β
GST	Glutathione S-transferases
H/R	Hypoxia/Reoxygenation
HMVEC	Human microvascular endothelial cell
I/R	Ischemia/Reperfusion
IL-6	Interleukin-6
LC3BII	Light chain 3B II
LDH	Lactate dehydrogenase
LDL	Low-density lipoprotein
LPL	Lipoprotein lipase
MAP	Mean atrial pressure
MAPK	Mitogen-activated protein kinase
MCP-1	Monocyte chemoattractant protein-1
MDA	Malondialdehyde
MMP	Matrix metalloproteinase
NADPH	Nicotinamide adenine dinucleotide phosphate
NF-κB	Nuclear factor kappa B
NOX	NAPDH oxidase
NRCM	Neonatal rat cardiomyocytes
Nrf2	Nuclear factor erythroid 2-related factor 2
p-p38	Phosphorylated p38
PPAR-γ	Peroxisome proliferator-activated receptor gamma
ROS	Reactive oxygen species
Ser9	Serine 9
sICAM-1	Soluble intercellular adhesion molecule-1
SIRT1	Silent information regulator 1
Smad3	Suppressor of mothers against decapentaplegic 3
SOD	Superoxide dismutase
sVCAM-1	Soluble vascular cell adhesion molecule-1
TAC	Total antioxidant capacity
TNF-α	Tumor necrosis factor alpha
TUNEL	Terminal deoxynucleotidyl transferase biotin-dUTP nick end labeling
α-SMA	Alpha smooth muscle actin
β-MHC	Beta myosin heavy chain

## References

[1] WHO Cardiovascular Diseases. https://www.who.int/health-topics/cardiovascular-diseases#tab=tab_1.

[2] CDC Heart Disease Facts. https://www.cdc.gov/heartdisease/facts.htm.

[3] Chen X., Li X., Xu X., Li L., Liang N., Zhang L., Lv J., Wu Y.-C., Yin H. (2021). Ferroptosis and cardiovascular disease: Role of free radical-induced lipid peroxidation. Free Radic. Res..

[4] Hajar R. (2017). Risk Factors for Coronary Artery Disease: Historical Perspectives. Heart Views.

[5] Amiri M., Majid H.A., Hairi F., Thangiah N., Bulgiba A., Su T.T. (2014). Prevalence and determinants of cardiovascular disease risk factors among the residents of urban community housing projects in Malaysia. BMC Public Health.

[6] Maurya A.P., Chauhan J., Yadav D.K., Gangwar R., Maurya V.K., Egbuna C., Mishra A.P., Goyal M.R. (2021). Chapter 11—Nutraceuticals and their impact on human health. Preparation of Phytopharmaceuticals for the Management of Disorders.

[7] Brahmkshatriya P.P., Brahmkshatriya P.S., Ramawat K.G., Mérillon J.-M. (2013). Terpenes: Chemistry, Biological Role, and Therapeutic Applications. Natural Products: Phytochemistry, Botany and Metabolism of Alkaloids, Phenolics and Terpenes.

[8] Buckle J. (2016). Basic Plant Taxonomy, Basic Essential Oil Chemistry, Extraction, Biosynthesis, and Analysis. Clinical Aromatherapy: Essential Oils in Healthcare.

[9] da Silva L.B., Camargo S.B., Moraes R.D., Medeiros C.F., Jesus A.D., Evangelista A., Villarreal C.F., Quintans L.J., Silva D.F. (2020). Antihypertensive effect of carvacrol is improved after incorporation in beta-cyclodextrin as a drug delivery system. Clin. Exp. Pharmacol. Physiol..

[10] Hou N., Mai Y., Qiu X., Yuan W., Li Y., Luo C., Liu Y., Zhang G., Zhao G., Luo J.D. (2019). Carvacrol Attenuates Diabetic Cardiomyopathy by Modulating the PI3K/AKT/GLUT4 Pathway in Diabetic Mice. Front. Pharm..

[11] Liu Y., Wei J., Ma K.T., Li C.L., Mai Y.P., Qiu X.X., Wei H., Hou N., Luo J.D. (2020). Carvacrol protects against diabetes-induced hypercontractility in the aorta through activation of the PI3K/Akt pathway. Biomed. Pharm..

[12] Xu S., Ilyas I., Little P.J., Li H., Kamato D., Zheng X., Luo S., Li Z., Liu P., Han J. (2021). Endothelial Dysfunction in Atherosclerotic Cardiovascular Diseases and Beyond: From Mechanism to Pharmacotherapies. Pharmacol. Rev..

[13] Rajendran P., Rengarajan T., Thangavel J., Nishigaki Y., Sakthisekaran D., Sethi G., Nishigaki I. (2013). The vascular endothelium and human diseases. Int. J. Biol. Sci..

[14] Little P.J., Askew C.D., Xu S., Kamato D. (2021). Endothelial Dysfunction and Cardiovascular Disease: History and Analysis of the Clinical Utility of the Relationship. Biomedicines.

[15] Li Q., Atochin D., Kashiwagi S., Earle J., Wang A., Mandeville E., Hayakawa K., d’Uscio L.V., Lo E.H., Katusic Z. (2013). Deficient eNOS Phosphorylation Is a Mechanism for Diabetic Vascular Dysfunction Contributing to Increased Stroke Size. Stroke.

[16] Kostov K. (2021). The Causal Relationship between Endothelin-1 and Hypertension: Focusing on Endothelial Dysfunction, Arterial Stiffness, Vascular Remodeling, and Blood Pressure Regulation. Life.

[17] Soehnlein O., Libby P. (2021). Targeting inflammation in atherosclerosis—From experimental insights to the clinic. Nat. Rev. Drug Discov..

[18] Ali S.S., Ahmad W., Budin S.B., Zainalabidin S. (2020). Implication of dietary phenolic acids on inflammation in cardiovascular disease. Rev. Cardiovasc. Med..

[19] Sun X., Deng K., Zang Y., Zhang Z., Zhao B., Fan J., Huang L. (2021). Exploring the regulatory roles of circular RNAs in the pathogenesis of atherosclerosis. Vascul. Pharmacol..

[20] Hamid A.A., Aminuddin A., Anuar N.N.M., Mansor N.I., Ahmad M.F., Saleh M.S.M., Mokhtar M.H., Ugusman A. (2022). *Persicaria minor* (Huds.) Opiz Prevents In Vitro Atherogenesis by Attenuating Tumor Necrosis Factor-α-Induced Monocyte Adhesion to Human Umbilical Vein Endothelial Cells. Life.

[21] Mohd Nor N.A., Budin S.B., Zainalabidin S., Jalil J., Sapian S., Jubaidi F.F., Mohamad Anuar N.N. (2022). The Role of Polyphenol in Modulating Associated Genes in Diabetes-Induced Vascular Disorders. Int. J. Mol. Sci..

[22] Jubaidi F.F., Zainalabidin S., Taib I.S., Abdul Hamid Z., Mohamad Anuar N.N., Jalil J., Mohd Nor N.A., Budin S.B. (2022). The Role of PKC-MAPK Signalling Pathways in the Development of Hyperglycemia-Induced Cardiovascular Complications. Int. J. Mol. Sci..

[23] Zeng Y., Xiong Y., Yang T., Wang Y., Zeng J., Zhou S., Luo Y., Li L. (2022). Icariin and its metabolites as potential protective phytochemicals against cardiovascular disease: From effects to molecular mechanisms. Biomed. Pharmacother..

[24] Sapian S., Taib I.S., Latip J., Katas H., Chin K.Y., Mohd Nor N.A., Jubaidi F.F., Budin S.B. (2021). Therapeutic Approach of Flavonoid in Ameliorating Diabetic Cardiomyopathy by Targeting Mitochondrial-Induced Oxidative Stress. Int. J. Mol. Sci..

[25] Si L.Y., Ali S.A.M., Latip J., Fauzi N.M., Budin S.B., Zainalabidin S. (2017). Roselle is cardioprotective in diet-induced obesity rat model with myocardial infarction. Life Sci..

[26] Kim N.-H., Kang P.M. (2010). Apoptosis in Cardiovascular Diseases: Mechanism and Clinical Implications. Korean Circ. J..

[27] Qian W., Wang Z., Xu T., Li D. (2019). Anti-apoptotic effects and mechanisms of salvianolic acid A on cardiomyocytes in ischemia-reperfusion injury. Histol. Histopathol..

[28] Bae S., Yalamarti B., Kang P.M. (2008). Role of caspase-independent apoptosis in cardiovascular diseases. Front. Biosci..

[29] Hinderer S., Schenke-Layland K. (2019). Cardiac fibrosis—A short review of causes and therapeutic strategies. Adv. Drug Deliv. Rev..

[30] Gunawardena G. Isoprene Rule. https://chem.libretexts.org/@go/page/39398.

[31] Perveen S., Al-Taweel A. (2018). Terpenes and Terpenoids.

[32] Yadav N., Yadav R., Goyal A. (2014). Chemistry of Terpenoids. Int. J. Pharm. Sci.

[33] Santos M.R., Moreira F.V., Fraga B.P., Souza D.P.D., Bonjardim L.R., Quintans-Junior L.J. (2011). Cardiovascular effects of monoterpenes: A review. Rev. Bras. Farmacogn..

[34] Ashour M., Wink M., Gershenzon J. (2010). Biochemistry of Terpenoids: Monoterpenes, Sesquiterpenes and Diterpenes. Annual Plant Reviews Volume 40: Biochemistry of Plant Secondary Metabolism.

[35] Harborne J.B. (1988). Phytochemical Methods.

[36] Morrow G.W., Morrow G.W. (2016). 258The Shikimate Pathway: Biosynthesis of Phenolic Products from Shikimic Acid. Bioorganic Synthesis: An Introduction.

[37] Paduch R., Kandefer-Szerszeń M., Trytek M., Fiedurek J. (2007). Terpenes: Substances useful in human healthcare. Arch. Immunol. Exp..

[38] Breitmaier E. (2006). Terpenes: Flavors, Fragrances, Pharmaca, Pheromones.

[39] Wang G., Tang W., Bidigare R. (2005). Terpenoids as Therapeutic Drugs and Pharmaceutical Agents. Natural Products: Drug Discovery and Therapeutic Medicine.

[40] Tiwari G., Tiwari R., Sriwastawa B., Bhati L., Pandey S., Pandey P., Bannerjee S.K. (2012). Drug delivery systems: An updated review. Int. J. Pharm. Investig..

[41] Duchêne D., Wouessidjewe D., Ponchel G. (1999). Cyclodextrins and carrier systems. J. Control. Release.

[42] Ma W., Guo W., Shang F., Li Y., Li W., Liu J., Ma C., Teng J. (2020). Bakuchiol Alleviates Hyperglycemia-Induced Diabetic Cardiomyopathy by Reducing Myocardial Oxidative Stress via Activating the SIRT1/Nrf2 Signaling Pathway. Oxid. Med. Cell Longev..

[43] Wang Z., Gao L., Xiao L., Kong L., Shi H., Tian X., Zhao L. (2018). Bakuchiol protects against pathological cardiac hypertrophy by blocking NF-κB signaling pathway. Biosci. Rep..

[44] Zhang X., Li X., Wang C., Li H., Wang L., Chen Y., Feng J., Ali Alharbi S., Deng Y. (2021). Ameliorative effect of ferruginol on isoprenaline hydrochloride-induced myocardial infarction in rats. Environ. Toxicol..

[45] Li W., Cao J., Wang X., Zhang Y., Sun Q., Jiang Y., Yao J., Li C., Wang Y., Wang W. (2021). Ferruginol Restores SIRT1-PGC-1α-Mediated Mitochondrial Biogenesis and Fatty Acid Oxidation for the Treatment of DOX-Induced Cardiotoxicity. Front. Pharm..

[46] Lee Y.H., Lim W., Sung M.K. (2018). Carnosic Acid Modulates Increased Hepatic Lipogenesis and Adipocytes Differentiation in Ovariectomized Mice Fed Normal or High-Fat Diets. Nutrients.

[47] Wei Y.J., Xu H.J., Chen J.J., Yang X., Xiong J., Wang J., Cheng F. (2020). Carnosic acid protects against pressure overload-induced cardiac remodelling by inhibiting the AKT/GSK3 beta/NOX4 signalling pathway. Exp. Ther. Med..

[48] Zhang Q.L., Yang J.J., Zhang H.S. (2019). Carvedilol (CAR) combined with carnosic acid (CAA) attenuates doxorubicin-induced cardiotoxicity by suppressing excessive oxidative stress, inflammation, apoptosis and autophagy. Biomed. Pharmacother..

[49] Baradaran Rahimi V., Momeni-Moghaddam M.A., Chini M.G., Saviano A., Maione F., Bifulco G., Rahmanian-Devin P., Jebalbarezy A., Askari V.R. (2022). Carnosol Attenuates LPS-Induced Inflammation of Cardiomyoblasts by Inhibiting NF-κB: A Mechanistic in Vitro and in Silico Study. Evid.-Based Complement. Altern. Med..

[50] Ou S., Lv J., Peng L., Zhao J., Chi L. (2017). Carnosol promotes endothelial differentiation under H_2_O_2_-induced oxidative stress. Arch. Biol. Sci..

[51] Chen Y., Ba L., Huang W., Liu Y., Pan H., Mingyao E., Shi P., Wang Y., Li S., Qi H. (2017). Role of carvacrol in cardioprotection against myocardial ischemia/reperfusion injury in rats through activation of MAPK/ERK and Akt/eNOS signaling pathways. Eur. J. Pharm..

[52] Sadeghzadeh S., Hejazian S.H., Jamhiri M., Hafizibarjin Z., Sadeghzadeh S., Safari F. (2018). The effect of carvacrol on transcription levels of Bcl-2 family proteins in hypertrophied heart of rats. Physiol. Pharmacol..

[53] Bayatmakoo R., Rashtchizadeh N., Yaghmaei P., Farhoudi M., Karimi P. (2017). Thymol decreases apoptosis and carotid inflammation induced by hypercholesterolemia through a discount in oxidative stress. Crescent J. Med. Biol. Sci..

[54] El-Marasy S.A., El Awdan S.A., Hassan A., Abdallah H.M.I. (2020). Cardioprotective effect of thymol against adrenaline-induced myocardial injury in rats. Heliyon.

[55] Shih M.F., Pan K.H., Liu C.C., Shen C.R., Cherng J.Y. (2018). Treatment of beta-thujaplicin counteracts di(2-ethylhexyl)phthalate (DEHP)-exposed vascular smooth muscle activation, inflammation and atherosclerosis progression. Regul. Toxicol. Pharmacol..

[56] Xiao H., Liang S., Cai Q., Liu J., Jin L., Yang Z., Chen X. (2022). Hinokitiol Protects Cardiomyocyte from Oxidative Damage by Inhibiting GSK3β-Mediated Autophagy. Oxid. Med. Cell Longev..

[57] Khushboo P.S., Jadhav V.M., Kadam V.J., Sathe N.S. (2010). *Psoralea corylifolia* Linn.-”Kushtanashini”. Pharm. Rev..

[58] Lim S.H., Ha T.Y., Ahn J., Kim S. (2011). Estrogenic activities of *Psoralea corylifolia* L. seed extracts and main constituents. Phytomedicine.

[59] Ulubelen A., Topçu G., Atta ur R. (1997). Chemical and biological investigations of *Salvia* species growing in Turkey. Studies in Natural Products Chemistry.

[60] Ulubelen A. (2003). Cardioactive and antibacterial terpenoids from some *Salvia* species. Phytochemistry.

[61] Sánchez-Camargo A.d.P., Herrero M. (2017). Rosemary (*Rosmarinus officinalis*) as a functional ingredient: Recent scientific evidence. Curr. Opin. Food Sci..

[62] Liu P., Dong J. (2017). Protective effects of carnosic acid against mitochondria-mediated injury in H9c2 cardiomyocytes induced by hypoxia/reoxygenation. Exp. Med..

[63] Ghasemzadeh Rahbardar M., Hosseinzadeh H. (2020). Therapeutic effects of rosemary (*Rosmarinus officinalis* L.) and its active constituents on nervous system disorders. Iran. J. Basic Med. Sci..

[64] Li X., Zhang Q., Hou N., Li J., Liu M., Peng S., Zhang Y., Luo Y., Zhao B., Wang S. (2019). Carnosol as a Nrf2 activator improves endothelial barrier function through antioxidative mechanisms. Int. J. Mol. Sci..

[65] Seyedan A.A., Dezfoulian O., Alirezaei M. (2020). *Satureja khuzistanica* Jamzad essential oil prevents doxorubicin-induced apoptosis via extrinsic and intrinsic mitochondrial pathways. Res. Pharm. Sci..

[66] Shakeri F., Khazaei M., Boskabady M.H. (2018). Cardiovascular Effects of *Nigella sativa* L. and its Constituents. Indian J. Pharm. Sci..

[67] Llana-Ruiz-Cabello M., Gutiérrez-Praena D., Pichardo S., Moreno F.J., Bermúdez J.M., Aucejo S., Cameán A.M. (2014). Cytotoxicity and morphological effects induced by carvacrol and thymol on the human cell line Caco-2. Food Chem. Toxicol..

[68] Agus H.H., Patel V.B., Preedy V.R. (2021). Chapter 4—Terpene toxicity and oxidative stress. Toxicology.

[69] El-Sayed S.M., Mansour A.M., Abdul-Hameed M.S. (2016). Thymol and Carvacrol Prevent Doxorubicin-Induced Cardiotoxicity by Abrogation of Oxidative Stress, Inflammation, and Apoptosis in Rats. J. Biochem. Mol. Toxicol..

[70] Ahmed O.M., Galaly S.R., Raslan M., Mostafa M. (2020). Thyme oil and thymol abrogate doxorubicin-induced nephrotoxicity and cardiotoxicity in Wistar rats via repression of oxidative stress and enhancement of antioxidant defense mechanisms. Biocell.

[71] Nagoor Meeran M.F., Javed H., Al Taee H., Azimullah S., Ojha S.K. (2017). Pharmacological Properties and Molecular Mechanisms of Thymol: Prospects for Its Therapeutic Potential and Pharmaceutical Development. Front. Pharmacol..

[72] Yu Y.M., Chao T.Y., Chang W.C., Chang M.J., Lee M.F. (2016). Thymol reduces oxidative stress, aortic intimal thickening, and inflammation-related gene expression in hyperlipidemic rabbits. J. Food Drug Anal..

[73] Tanabe J., Takashima Y., Ishiguri F., Sanpe H., Ohshima J., Iizuka K., Yokota S. (2019). Differences in β-thujaplicin content of wood between plantation- and naturally grown *Thujopsis dolabrata* var. *hondae* (hinokiasunaro) trees in Shimokita Peninsula, Aomori, Japan. J. Wood Sci..

[74] Jayakumar T., Hsu W.-H., Yen T.-L., Luo J.-Y., Kuo Y.-C., Fong T.-H., Sheu J.-R. (2013). Hinokitiol, a Natural Tropolone Derivative, Offers Neuroprotection from Thromboembolic Stroke In Vivo. Evid. Based Complement. Altern. Med..

[75] University of California D. Comparison of the Cosmetic Effects of Bakuchiol and Retinol. https://ClinicalTrials.gov/show/NCT03112863.

[76] Instituto Venezolano de Investigaciones C. Study to Verify the Effectiveness and Safety of Isothymol or Carvacrol Compound against SARS-CoV-2 in COVID-19 Patients. https://ClinicalTrials.gov/show/NCT05445089.

[77] Centro Universitario de Ciencias de la Salud M. Thymol on Netrin-1 on Obese Patients. https://ClinicalTrials.gov/show/NCT05427721.

